# Novel Actions of Growth Hormone in Podocytes: Implications for Diabetic Nephropathy

**DOI:** 10.3389/fmed.2017.00102

**Published:** 2017-07-12

**Authors:** Dhanunjay Mukhi, Rajkishor Nishad, Ram K. Menon, Anil Kumar Pasupulati

**Affiliations:** ^1^Department of Biochemistry, School of Life Sciences, University of Hyderabad, Hyderabad, India; ^2^Department of Pediatric Endocrinology and Physiology, University of Michigan, Ann Arbor, MI, United States

**Keywords:** growth hormone, podocytes, diabetic nephropathy, zinc finger E-box binding homeobox2, dedifferentiation, hypertrophy

## Abstract

The kidney regulates water, electrolyte, and acid-base balance and thus maintains body homeostasis. The kidney’s potential to ensure ultrafiltered and almost protein-free urine is compromised in various metabolic and hormonal disorders such as diabetes mellitus (DM). Diabetic nephropathy (DN) accounts for ~20–40% of mortality in DM. Proteinuria, a hallmark of renal glomerular diseases, indicates injury to the glomerular filtration barrier (GFB). The GFB is composed of glomerular endothelium, basement membrane, and podocytes. Podocytes are terminally differentiated epithelial cells with limited ability to replicate. Podocyte shape and number are both critical for the integrity and function of the GFB. Podocytes are vulnerable to various noxious stimuli prevalent in a diabetic milieu that could provoke podocytes to undergo changes to their unique architecture and function. Effacement of podocyte foot process is a typical morphological alteration associated with proteinuria. The dedifferentiation of podocytes from epithelial-to-mesenchymal phenotype and consequential loss results in proteinuria. Poorly controlled type 1 DM is associated with elevated levels of circulating growth hormone (GH), which is implicated in the pathophysiology of various diabetic complications including DN. Recent studies demonstrate that functional GH receptors are expressed in podocytes and that GH may exert detrimental effects on the podocyte. In this review, we summarize recent advances that shed light on actions of GH on the podocyte that could play a role in the pathogenesis of DN.

## Introduction

**Figure 1:**
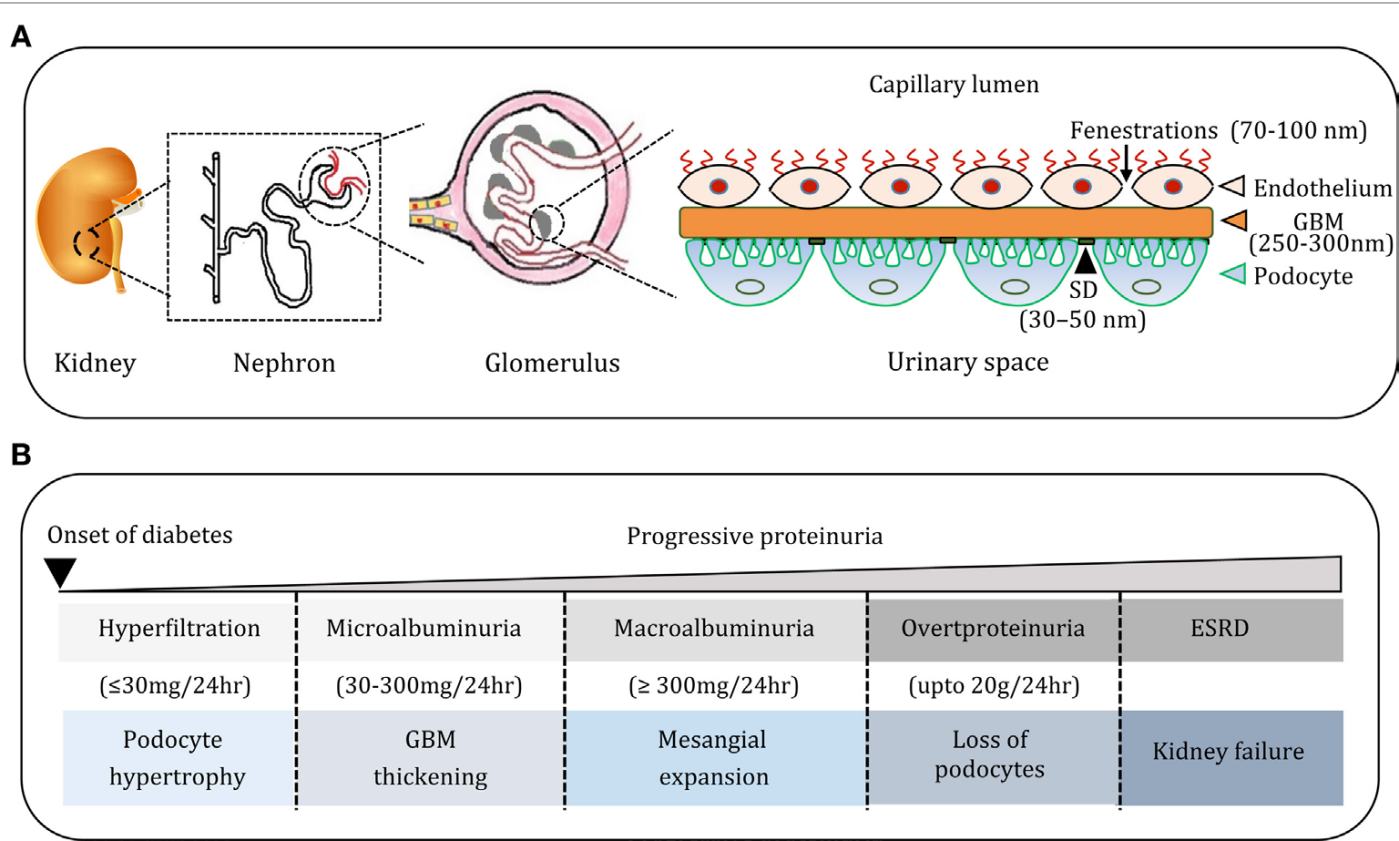
**(A)** Cartoon depicting the organization of nephron, glomerulus, and glomerular filtration barrier. The glomerular filtration barrier (GFB) comprises endothelial cells (ECs) of glomerular capillaries, basement membrane (BM), and podocytes (PC). Podocytes are specialized glomerular epithelial cells and slit diaphragm (SD) forms a contact between them and contributes to the glomerular permselectivity. **(B)** The natural course of diabetic nephropathy (DN). Progression of DN is associated with progressive proteinuria from micro albuminuria to overt proteinuria. The course of DN is also associated with histological manifestations in the glomerulus such as hypertrophy of podocytes, widening of glomerular BM, glomerulosclerosis, and depletion of podocytes, and these events culminate in end-stage renal disease.

The vertebrate kidney plays an essential role in filtration of blood, regulation of water, electrolyte, and acid-base balance, and thereby maintenance of overall body homeostasis. The function of the kidney to ensure almost protein-free ultrafiltered urine depends on the collective action of millions of nephrons (1). A nephron comprises two highly coordinated units: glomerulus and renal tubule. The glomerulus filters plasma to prevent protein loss into the glomerular filtrate. The renal tubule reabsorbs water and electrolytes in addition to contributing selective salts and Tamm–Horsfall proteins to the glomerular filtrate. The contribution of renal tubular absorption and secretion notwithstanding, the final composition of urine is largely determined by the integrity of glomerular filtration barrier (GFB, Figure 1A). The GFB consists of three critical components—endothelium, glomerular basement membrane (GBM), and podocytes (1, 2). The endothelium lines the inner surface of glomerular capillaries and is fenestrated (70–100 nm), which allows for small molecules and a limited amount of protein to pass through. The GBM (250–300 nm) is an extracellular matrix (ECM) material made up of structural proteins, the most abundant being collagen, fibronectin, and heparan sulfate proteoglycans. Podocytes are terminally differentiated visceral cells that adhere firmly to GBM and offer epithelial coverage to the surface of glomerular capillaries (1, 2). The slit pore between neighboring podocytes is 30–50 nm wide (3). This three-layered GFB with a distinct architecture serves as a size-selective and charge-dependent molecular sieve that facilitate the filtration of water, electrolytes, and small solutes while restricting the passage of negatively charged macromolecules including proteins and polypeptides. Proteins with a molecular weight of up to 20 kDa filter easily across the GFB (4). However, the smaller proteins are largely reabsorbed by proximal convoluted tubule, and only small amounts of protein are excreted. When the nephron function is disturbed, varying amounts of plasma proteins, particularly albumin (~67 kDa), are excreted in urine (Figure 1B) and albuminuria is a well-known index of adverse renal function (5).

Proteinuria can be classified as glomerular, tubular, and overflow (6). Glomerular disease is the most common cause of pathologic proteinuria that presents with large protein losses (>4 g per 24 h). Glomerular diseases that elicit proteinuria include minimal change disease (MCD), focal segmental glomerulonephritis (FSGS), membranous proliferative glomerulonephritis, IgA nephropathy, DN, collagen vascular disorders, amyloidosis, preeclampsia, and certain infections. Since GFB is the main constituent of the renal filtration apparatus, the appearance of protein in urine can be explained by damage to GFB, particularly to the podocytes as they play a key role in ensuring permselectivity of GFB. Tubular proteinuria is caused by tubulointerstitial disease results from decreased tubular reabsorption of proteins from the glomerular ultrafiltrate. In the case of overflow proteinuria, low-molecular-weight proteins overwhelm the ability of the proximal tubules to reabsorb proteins from the glomerular ultrafiltrate. Most often, this is a result of the immunoglobulin overproduction that occurs in multiple myeloma. In general, in tubular and overflow proteinuria less than 2 g of protein is excreted in 24 h.

Podocytes have a unique architecture consisting of a cell body with major and minor processes that extend outward from the cell body and interdigitated foot processes (FPs) that enwrap the glomerular capillaries (7, 8). Adjacent FPs are connected with slit diaphragm (SD) that forms the sole contact between podocytes. The extracellular domains of several transmembrane proteins such as nephrin, Neph-1, podocin, P-cadherin, and CD2AP constitute the SD. The unique structure and localization of podocytes enable them to oppose hydrostatic pressure within the glomerular capillaries and ensure glomerular filtration. Since podocytes remain attached to the GBM by only their FPs, they are always in danger of becoming detached and excreted in the urine (9). Furthermore, podocytes are continually exposed to metabolic waste products, drugs, and toxins that are routed to the kidneys for elimination. Podocyte injury is observed in both diabetic and non-diabetic renal diseases. The spectrum of podocyte diseases includes MCD, FSGS, collapsing glomerulonephropathy, inflammatory glomerulonephropathy, and DN, to name a few (10, 11).

## Podocyte Injury During Diabetes

It is generally considered that podocytes are terminally differentiated visceral epithelial cells of the glomerulus with no or limited proliferating capacity. Hence, the loss of a podocyte cannot be compensated by regeneration of a podocyte from neighboring healthy podocytes. Morphologically podocyte injury is manifested by distortion and fusion of FPs, which leaves the GBM uncovered and such injury is termed as effacement (12). Either injury or loss of podocytes is an early event in the pathology of DN in both humans and animal models. Podocytes are vulnerable to various insults during diabetes that result in apoptosis or dedifferentiation. Another noticeable feature of podocyte injury is hypertrophy by which podocytes could compensate for the loss of neighboring podocytes. Adaptive hypertrophy of podocytes in response to the loss of neighboring podocytes has limited effectiveness since these hypertrophic podocytes have to cover increased area of filtration surface that could decrease adhesiveness to the GBM (13). Also, it is hypothesized that hypertrophied podocytes are more vulnerable to prevailing stress conditions (13–15). Progressive loss of podocytes is one of the hallmarks of glomerulosclerosis (16).

Aberrations in various cellular and molecular events mediate podocytopathy during diabetes including hyperglycemia-induced increased ROS production, advanced glycation end products formation, aberrant Notch signaling, elevated vascular endothelial growth factor and transforming growth factor type β (TGF-β) signaling, and inappropriate activation of the renal renin–angiotensin system (1). Increased concentrations of angiotensin II in diabetes mellitus (DM) induces podocyte apoptosis by enhancing the expression of transient receptor potential cation channel 6 and this effect is reduced by inhibiting the nuclear translocation of nuclear factor kappa-light-chain-enhancer of activated B cells (NF-kB) (17, 18). TGF-β transgenic mice develop progressive mesangial expansion, which is associated with podocyte apoptosis through activation of mitogen-activated protein kinase (MAPK) dependent caspase-3 pathway (19). Growth factors elicit glomerular hypertrophy by acting directly on podocytes and/or on the other glomerular cells such as endothelial and mesangial cells. In addition to these signaling molecules and cytokines, endocrine mediators can also exert an adverse effect on podocytes. Hence, elevated levels of growth hormone (GH) are implicated in the early renal hypertrophy and proteinuria in type 1 DM (T1DM) (20). Excess GH secretion is a feature of poorly controlled T1DM and is posited to be a causative factor in the development of microangiopathic complications of diabetes including retinopathy and nephropathy (Table 1). The focus of this review is to summarize the novel actions of GH in the pathogenesis of podocyte injury in T1DM.

**Table 1:** Significant findings in growth hormone (GH)-mediated podocytopathy and diabetic nephropathy (DN).

Key findings of GH in podocyte biology	Reference
Identification of GH receptor (GHR) on podocytes	(56)
GH induces zinc finger E-box binding homeobox2 expression causes dedifferentiation and detachment of podocytes	(71)
GH reduces P-cadherin expression and alters podocyte permeability to albumin	(71)
GH induces transforming growth factor beta induced protein expression and promotes podocyte apoptosis	(81)
GH transgenic mice showed significant podocyte hypertrophy	(91)
GHR knockout diabetic mice protected from development of DN	(55)
Loss of binucleated and multinucleated podocytes in urine	(65)
GH activates mTOR pathway	(95)
mTOR overactivation recapitulates features of DN	(92, 93)

## Altered GH/GH Receptor (GHR) Axis and Renal Dysfunction

**Figure 2:**
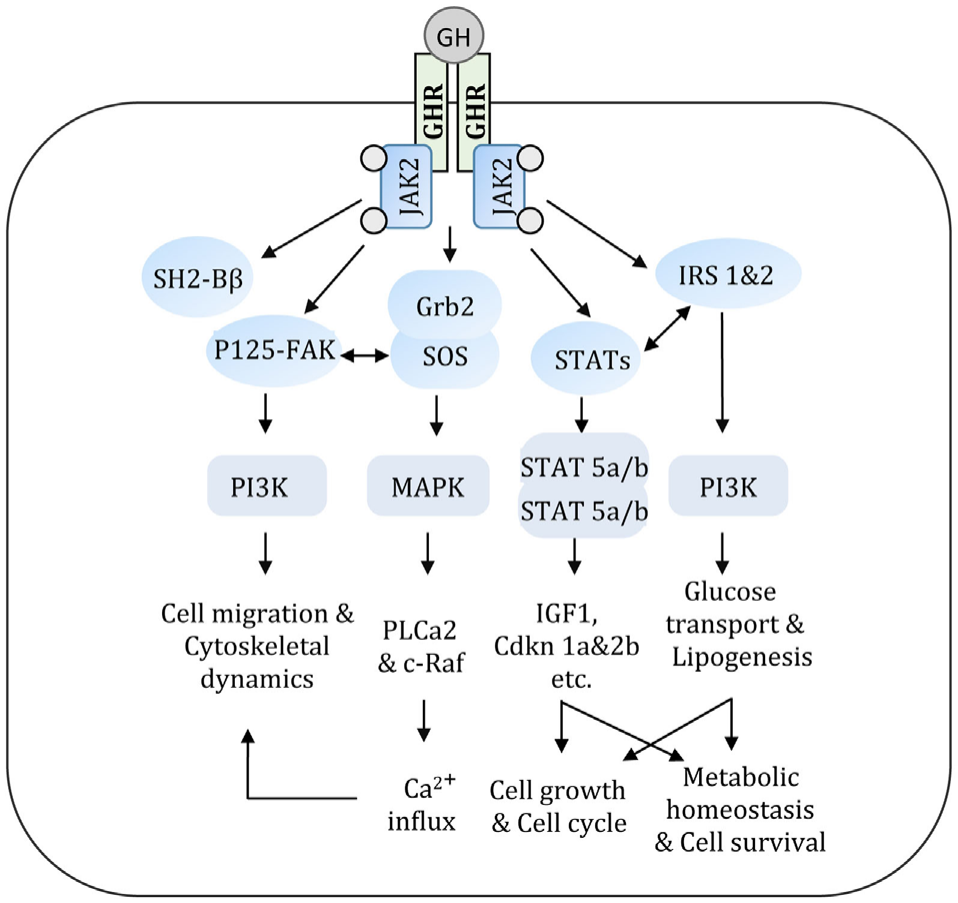
GH acts *via* a variety of signal transduction pathways. Multiple GH signaling pathways can contribute to specific GH responses. GH binds to GHR and activates JAK2 that in turn triggers an array of signaling cascade. These interconnected signal transduction pathways regulate various metabolic and cellular events. GH, growth hormone; GHR, growth hormone receptor; JAK2, Janus kinase 2; IRS, insulin receptor substrate; PI3K, phosphatidyl inositol 3-kinase; STAT, signal transducer and activator of transcription; Grb2-SOS, growth factor receptor-bound 2-son of sevenless complex; FAK, focal adhesion kinase; SH2-Bβ, src-homology 2 domain Bβ; MAPK, mitogen-activated protein kinase.

Human GH is synthesized by somatotrophs of the anterior pituitary gland and stimulates postnatal growth, cell proliferation, and regeneration (21). Secretion of GH from the pituitary is primarily controlled by GH-releasing hormone and somatostatin, where former induces GH secretion and later inhibits GH secretion. GH elicits its actions at the cellular, molecular, and organ levels *via* interacting with GHR. Binding of GH dimerizes GHR and induces a conformational change in its cytoplasmic portion, which allows activation of Janus kinase 2 (JAK2) (22). GH-mediated cell signaling events are summarized in Figure 2. Autophosphorylation of JAK2 by GHR facilitates the activation of downstream signaling proteins including signal transducers and activators of transcription (STATs), MAPK, insulin receptor substrate (IRS-1&2), and phosphatidylinositol 3-kinase (22–25). Upon activation (phosphorylation), STAT proteins translocate to the nucleus and stimulate transcription of an array of GH-regulated genes. The JAK2-STAT pathway is regulated by suppressor of cytokine signaling proteins. The association of growth factor receptor-bound 2-son of sevenless complex with JAK2 is critical for the activation of MAPK pathway by GH (26, 27). GH potentially exerts its actions directly on target cells and indirectly by stimulating the production of insulin-like growth factor (IGF-1) (28). An intact JAK2–STAT5b signaling pathway is essential for stimulating the production of IGF-1. Circulating levels of IGF-1 regulate GH secretion by the anterior pituitary *via* a negative feedback loop and is used in clinical medicine as a surrogate marker of GH action (29). Although GH induces IGF-1 synthesis and secretion by hepatocytes, the rate of IGF-1 clearance and levels of IGF-binding proteins (IGFBPs) in blood also determine the concentrations of free IGF-1 at the tissue level. Several components of GH/GHR axis, including GHR, IGF-1, IGF-1 receptor, and IGFBPs are expressed in the kidney with precise spatial distribution across various segments of the nephron (30, 31). GH and IGF-1 have significant effects on renal function by regulating cellular hyperplasia and, hypertrophy, intrarenal blood flow, and tubular reabsorption (2, 32, 33). GH signaling regulates the intrarenal hemodynamics and glomerular arteriolar vasodilation by inducing cyclooxygenase activity and generation of nitric oxide (2, 34).

**Figure 3:**
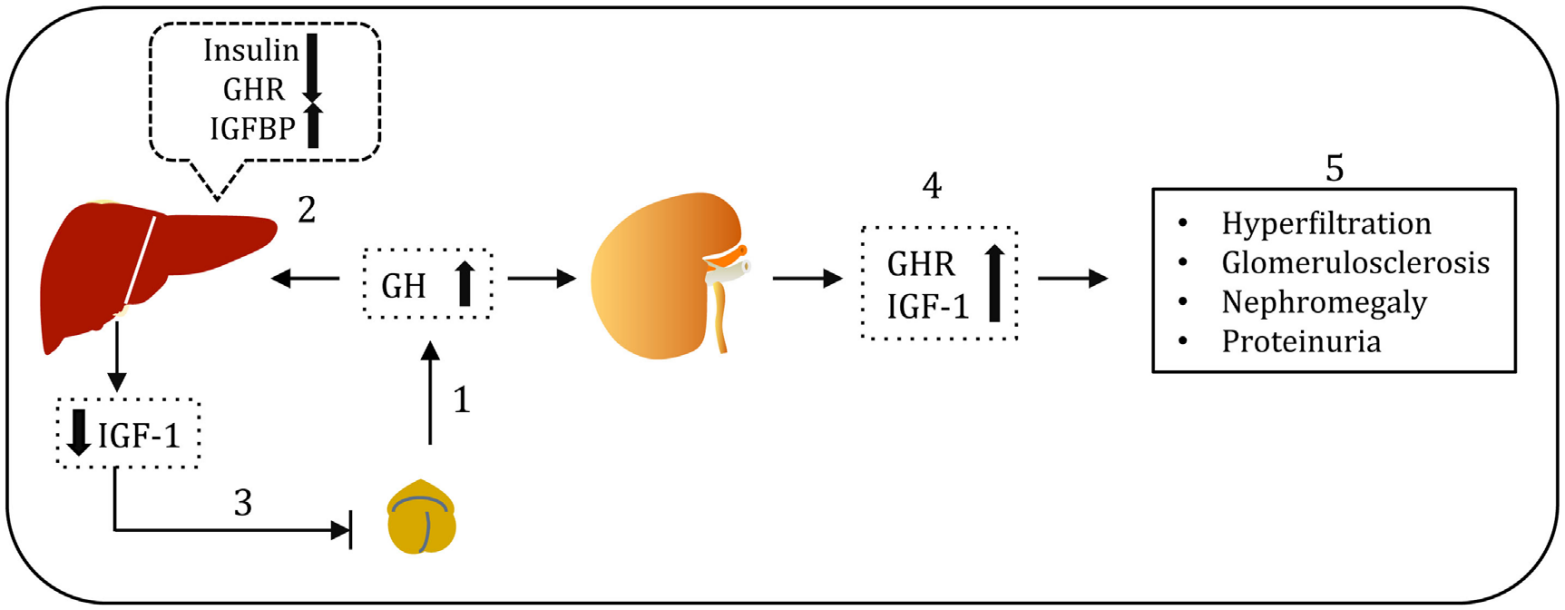
Growth hormone (GH)–GH receptor (GHR)–IGF-1 axis in type 1 diabetes. Pituitary GH acts *via* GHR and induces hepatic insulin-like growth factor (IGF-1) production, which serves as a surrogate marker for GH action (1). However, in type 1 diabetes, reduced portal insulin levels results in decreased expression of hepatic GHR, impaired IGF-1 production and elevated IGFBP-1 levels (2). Low bioavailability of IGF-1 leads to compensatory GH hypersecretion *via* negative feedback loop mechanism (3). Except in liver, GHR expression in other tissues including kidney is not compromised. Elevated GH levels in poorly controlled type 1 diabetes associated with elevated GHR and IGF-1 in the kidney (4). Elevated GH levels are implicated in the renal hyperfiltration, glomerulosclerosis, nephromegaly, and proteinuria (5).

Alterations in the GH/GHR axis have been described in T1DM and diabetic kidney diseases (2). The mean 24 h concentration of circulating GH is elevated in poorly controlled T1DM. Elevation of GH levels in diabetes can be explained by two interrelated mechanisms (Figure 3). In poorly controlled T1DM, decreased hepatic GHR expression results in GH resistance and consequent attenuation of hepatic IGF-1 production (35). The resulting low levels of circulating IGF-1 stimulate GH secretion by feedback mechanism (36). Additionally, hypoinsulinemia in T1DM results in increased hepatic production of IGFBPs (37). The increase in serum IGFBPs, in particular IGFBP-1, dampens IGF-1 action at the cellular level and thus feedbacks on the pituitary somatotroph to stimulate GH secretion (38, 39). However, in contrast to the liver, GHR expression in the kidney is unaltered or even increased in poorly controlled DM, thus exposing the kidney to the effects of elevated levels of GH (40). Administration of GH for a week to healthy volunteers resulted in increased glomerular filtration rate (GFR) (41, 42). On the other hand, administration of somatostatin analogs: octreotide (SMS 201-995) and somatulin, which serves as inhibitors of GH, attenuated GFR, renal hyperfiltration, and kidney size in subjects with T1DM (43, 44). A study of a cohort of patients with acromegaly (a condition resulting from excessive GH levels secondary to a pituitary adenoma) demonstrated adverse effects of elevated GH levels on renal structure and function (45). Microalbuminuria is significantly increased in patients with acromegaly compared to healthy volunteers. Surgical ablation of pituitary adenoma results in decreased GH levels and kidney size (46). An increase in the GFR and kidney size was observed in both humans and rats injected with GH (47, 48). A 25–50% elevation in the GFR was observed in early course of T1DM (49). It was reported that glomerular hypertrophy and increased kidney size typically accompany the rise in GFR (50). Hyperfiltration, increased renal plasma flow, and hypertrophy that are induced by GH administration may accelerate the renal injury (51, 52). However, the clinical relevance of increase in GFR with GH administration is not precisely known. Elevated levels of GH in transgenic mice have been linked to diabetic kidney disease and other renal complications (53, 54). On the other hand, mice with a disrupted GHR gene or those expressing GH antagonist are protected from glomerular hypertrophy (55). Although these studies highlight the importance of GH/GHR axis in renal biology and the association of overactivity of GH signaling with the adverse renal outcome, the precise molecular mechanism(s) of GH action on podocytes remained uncertain until the recent identification of GHR in glomerular podocytes (56).

## Identification of GHR in Podocytes

It had been known for some time that increased levels of circulating GH was associated with glomerular hypertrophy, hyperfiltration, and proteinuria (57, 58). However, it was not clear whether these effects of GH were due to a direct action of GH on various cell types of the glomerulus or an indirect effect secondary to GH’s effects on hemodynamic parameters such as blood pressure and vascular tone (59). A significant advancement in this field was the identification of GHR in mouse and human podocytes with activation of canonical JAK–STAT signaling cascade and MAP kinase pathways in a GH-dependent manner (56). Exposure of podocytes to GH resulted in intracellular redistribution of the JAK2 adapter protein SH2-Bβ, activation of focal adhesion kinase, production of reactive oxygen species, and GH-dependent remodeling of actin cytoskeleton (56). These findings provide evidence for a direct effect of GH on podocytes. Besides podocytes, the GHR is also expressed in mesangial cells, proximal tubule, ascending limb of Henle’s loop, and collecting ducts (60–62). The expression of GHR exhibits a decreasing gradient from the renal cortex to the medulla. GHR levels are approximately 10–20 times higher in the proximal tubule than in downstream segments of the renal tubule.

## GH Induces Dedifferentiation of Podocytes

**Figure 4:**
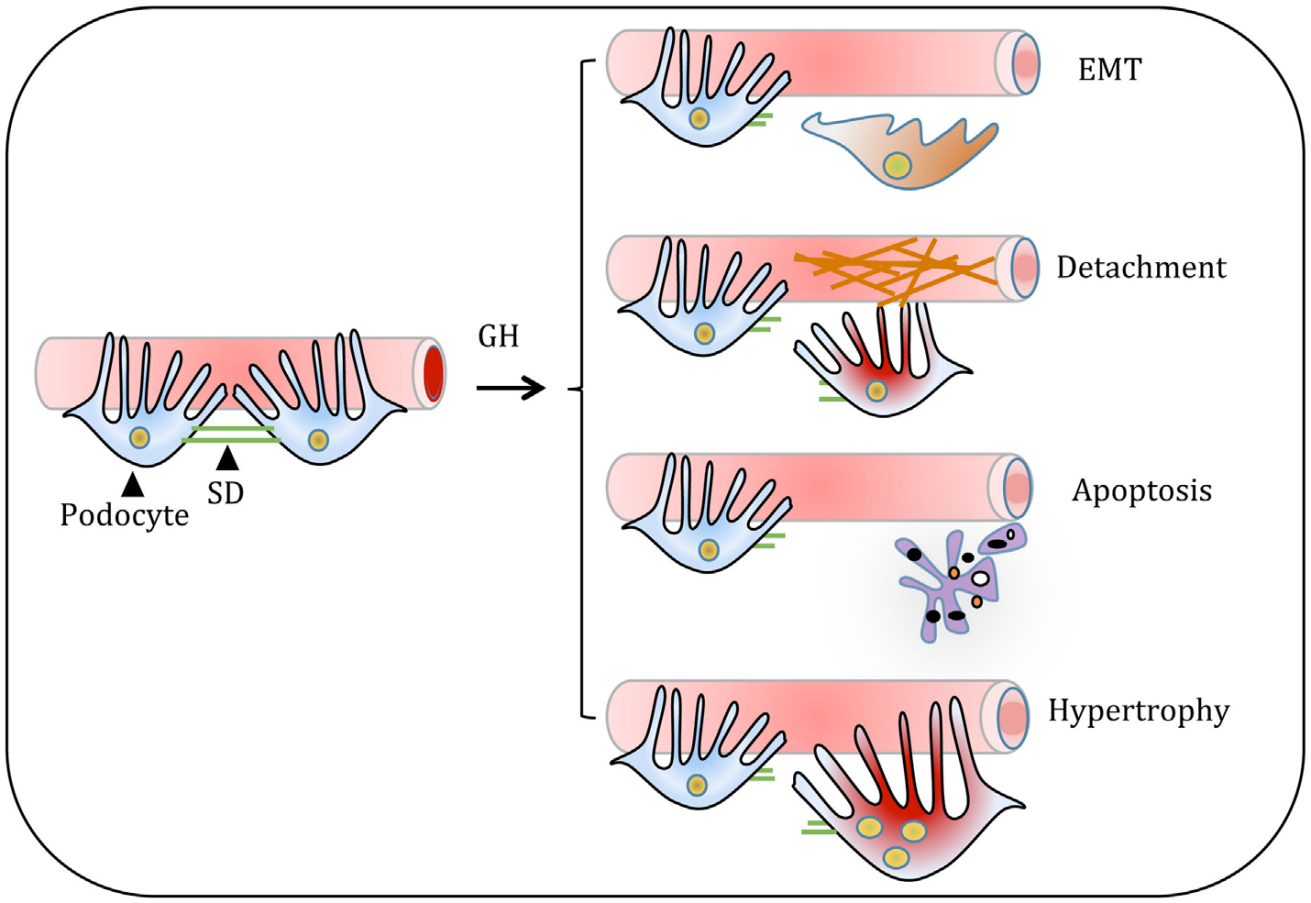
Proposed model for the growth hormone (GH) action on glomerular podocyte. GH-induced cellular events in podocyte include dedifferentiation of podocytes or thickening and/or cross-linking of the basement membrane. Both these events result in the shedding of podocytes. Alternatively, GH induces podocyte apoptosis and hypertrophy. All these changes in podocytes manifest in decreased podocyte count and impair glomerular function in pathological states of overactivity of GH/GH receptor axis such as acromegaly and type 1 diabetes.

Podocyte loss has been reported in IgA nephropathy, HIV-associated nephropathy, and DN. Several studies have reported the presence of podocytes in urine sediments of patients with glomerular diseases supporting the argument that podocytes detach from GBM (63, 64). It was reported that patients with glomerular diseases shed viable podocytes in urine (65). Interestingly, these podocytes isolated from urine of the patients with the glomerular disease can be cultured under suitable conditions (65). The presence of viable podocytes in urinary sediments suggests that these podocytes could detach from basement membrane (BM) into urinary space (Figure 4).

Podocytes originate from columnar epithelial cells, which in turn arise from the metanephric mesenchyme *via* a process termed as mesenchymal–epithelial transition (MET). Columnar epithelial cells possess apical tight junctions and adherent junctions, which migrate basally and transform into the SD during podocyte morphogenesis (66). It is reasonable to posit that in glomerular diseases mature podocytes undergo dedifferentiation that mimics the reversal of MET. Epithelial cells are fully differentiated and immobile having cell–cell junctions and cell–matrix interactions. Epithelial cells express several adhesion molecules such as E-cadherin, epithelial cell adhesion molecule, and tight junction proteins including occludins and junction adhesion molecules. Unlike epithelial cells, mesenchymal cells are motile and invasive and express a distinct set of markers such as vimentin, snail, slug, and N-cadherin (67). Epithelial cells upon undergoing dedifferentiation detach from the substratum by disrupting cell–cell contacts and cytoskeletal rearrangements and attain migratory properties (68) Podocyte dedifferentiation during injury is evidenced by loss of epithelial polarity, loss of cell–cell junctions, and increase in mesenchymal characteristics (69). The process of podocyte dedifferentiation recapitulates some of the features with a fundamental cell remodeling mechanism known as epithelial–mesenchymal transition (EMT) that occurs during embryonic development and wound healing. EMT has been categorized into three types based on their origin: type 1, associated with embryogenesis and organ development; type 2, associated with wound healing and fibrosis; and type 3, associated with cancer cell invasion, migration, and metastasis. The mechanism of podocyte dedifferentiation resembles type 2 EMT (69, 70).

Recent studies shed light on the mechanisms of GH-mediated dedifferentiation in podocytes. Studies from our group revealed that in both animal models and cultured podocytes, GH induces the expression of transcription factor zinc finger E-box binding homeobox2 (ZEB2) (71). ZEB2 orchestrates dedifferentiation of epithelial cells by promoting a cadherin switch—decreasing expression of E- and P-cadherin with a concomitant increase in expression of N-cadherin (71). It was further identified that GH induces the expression of a natural antisense transcript specific to ZEB2 (ZEB2-NAT). ZEB2-NAT, synthesized from 5′UTR of *ZEB2* gene in an antisense manner, specifically binds to a complementary region located in the internal ribosomal entry site (IRES) of ZEB2 mRNA (72). The complimentary base pairing of ZEB2-NAT to the ZEB2 mRNA prevents splicing of an IRES element, which in turn facilitates increased translation of ZEB2 mRNA (71). Thus, GH induces dedifferentiation of podocytes by regulating both ZEB2 and ZEB2-NAT. The dedifferentiation has significant consequences on the function of the podocyte, particularly on the epithelial cell–cell junctions: expression of SD is lost and FPs are effaced in the diseased podocytes. It is noteworthy that a recent study has elucidated the role of the microRNA 96-182-183 cluster in GH-dependent cell transition and invasion in breast cancer (73).

## GH Promotes Accumulation of ECM Components

Extracellular matrix offers mechanical strength and provides signals for differentiation and tissue morphogenesis. The components of ECM include matrix polysaccharides, structural proteins, and interlinking proteins. The most abundant matrix polysaccharides of ECM are glycosaminoglycans, hyaluronan, and heparan sulfates, which aggregate with linker proteins to form larger complex proteoglycans. Proteoglycans such as agrin and perlecan offer negative charge throughout the GBM (74). Negative charges on the GBM offer electrostatic repulsion to anionic molecules and impede the flow of these molecules into the glomerular filtrate (75). Collagen type IV, type XVII, and nidogen are most prevalent matrix structural proteins of the GBM. Laminin and fibronectin are matrix interlinking proteins known to interact with proteoglycans and collagen. Matrix metalloproteases (MMPs), serine, and cysteine proteases are classes of enzymes that degrade ECM proteins. Tissue inhibitors of metalloproteases (TIPMs) are the known inhibitors of MMPs that are upregulated in the early pathogenesis of DN (76). Together TIPMs and MMPs maintain ECM homeostasis. Excess synthesis and deposition with decreased clearance of ECM proteins in the glomerulus result in glomerulosclerosis.

During DN, accumulation of ECM proteins predominantly occurs in the glomerular mesangium, tubulointerstitium, and GBM. Glomerular endothelial cells (ECs) and podocytes mainly dictate the composition of GBM (77). Mass spectrometry-based proteomics approach revealed that both glomerular ECs and podocytes synthesize more than 50% of ECM proteins that comprises the core of the ECM (77). However, the mechanism(s) for increased expression of ECM proteins during DN is not well understood. Increased deposition of ECM proteins, including collagen type IV, laminin B2, and heparan sulfate proteoglycans, in GH transgenic mice resulted in progressive glomerulosclerosis (53, 78). Studies with GH transgenic mice, experiments with cultured podocytes, and rats injected with GH have provided evidence that GH promotes synthesis and accumulation of ECM components (79). Thickening of GBM and mesangial sclerosis was observed in glomerular sections of rats administered with GH (79, 80). GH administration to diabetic rats showed further increase (30%) in glomerulosclerosis and tubulointerstitial fibrosis (80). A significant decrease in the expression of MMP2 and MMP9 was observed with GH treatment in streptozotocin-induced diabetic male rats (80). MMP2 and MMP9 are the major matrix proteases associated with ECM turnover. In our earlier study, we employed ECM and adhesion molecules profiler PCR array and analyzed the effect of GH on the expression of 84 genes that are important for cell–cell and cell–matrix interactions (81). This array contains ECM proteins including BM constituents, collagens, and other genes defining ECM structure. GH induced the expression of collagen type II α1, integrin E, laminin subunit α3, and laminin subunit β3 (81). The data suggest that GH promotes increased synthesis of ECM components. Coincident with the synthesis of ECM components, GH treatment resulted in reduced expression of MMPs, such as MMP7 and MMP13-15, which degrade extracellular molecules (81). We observed elevated expression of transforming growth factor beta-induced protein (TGFBIp) in podocytes treated with GH (81). TGFBIp is also induced by TGF-β and inhibit cell adhesion and interacts with collagen and other matrix structural proteins including integrins. Overall, these studies indicate that GH increases the synthesis of ECM components and contributes to their cross-linking and stiffness. These effects of GH impede the adhesion of podocytes to the GBM resulting in loss of podocytes (Figure 4).

## GH Induces Podocyte Apoptosis

Unlike other cell types of the glomerulus, podocytes are postmitotic, terminally differentiated cells, which can replicate DNA but do not undergo proliferation because they arrest in the G0/G1 phase of cell cycle (82). Podocyte depletion is well documented in DN and apoptosis of podocyte is considered as one of the mechanisms for decreased podocyte count (83). Podocytes undergo apoptosis at early stages in the course of progressive glomerulosclerosis and podocyte loss precedes mesangial expansion. Several nutrients, cytokines, and growth factors differentially contribute to podocyte viability. Insulin and IGF-1 are critical for podocyte survival (84, 85), whereas high concentrations of glucose and TGF-β are known to induce podocyte apoptosis (19, 86).

Studies from our group demonstrated that mouse podocytes exposed to GH undergo apoptosis (81). A time-dependent increase in the rate of GH-induced apoptosis of mouse podocytes was observed (81). GH-induced podocyte apoptosis can be explained in two possible ways. GH induces reactive oxygen species (ROS) in podocytes and these ROS are implicated in cell death (56, 61). Quenching of GH-induced ROS by *N*-acetyl cysteine prevented GH-mediated podocyte cell death (56). Alternatively, GH-induced TGFBIp is also implicated in podocyte cell death (81). Podocytes exposed to exogenous TGFBIp underwent apoptosis (81). Furthermore, TGFBIp-induced apoptosis has been reported in other renal cell types including renal tubular epithelial cells and retinal ECs (87, 88). Anti-TGFBIp antibody inhibited apoptosis that was induced by exogenous TGFBIp (88). These studies suggest that GH induces podocyte apoptosis that could contribute to decreased podocyte density in conditions, such as poorly controlled diabetes, with elevated concentrations of GH (81).

## The Role of GH/GHR Axis in Podocyte Hypertrophy

Renal hypertrophy and enlarged glomeruli are prominent features during the early course of T1DM. This glomerular enlargement is associated with podocyte hypertrophy rather than hyperplasia (Figure 4) (89). It is postulated that hypertrophy of podocytes is the compensatory cellular response for the loss of neighboring podocytes. In addition to compensating for the loss of podocyte function, the podocyte enlargement may also help to cover the areas of on glomerular capillaries that have been exposed because of detachment and loss of podocytes. These advantages notwithstanding the hypertrophic response could be a temporary adaptive response, which could ultimately result in detachment and shedding of podocytes in the urine. Recovery of viable and cultivable podocytes from the urine of subjects with DN supports such a scenario (90). It is noteworthy that 10–50% of viable podocytes in the urinary sediments are multinucleated and can be maintained for several days in *in vitro* conditions (65). The presence of multinucleated podocytes suggests impaired coordination between karyokinesis and cytokinesis.

Elevated GH levels are associated with renal hypertrophy (91). We have observed an increase in kidney size in rats injected with GH for 7 days. Increase in kidney size in streptozotocin-induced diabetes is ameliorated in mice where GHR in podocytes is knocked out. However, the cellular mechanism(s) regulating podocyte hypertrophy remains unclear. Mammalian target of rapamycin complex1 (mTORC1) regulates cell size. mTORC1 is a major cell survival kinase activated by Akt and inhibited by tuberous sclerosis complex (TSC1/2) (92, 93). Constitutive activation of mTORC1 recapitulated several DN features including podocyte hypertrophy, proteinuria, and glomerular sclerosis (92). Inhibition of podocyte-specific mTORC1 prevents podocyte hypertrophy and progressive glomerulosclerosis during diabetes (93, 94). GH potentially stimulates protein synthesis by activation of mTOR kinase in human cell lines (95). Mice transgenic for GH and null to IGF1 showed substantial podocyte hypertrophy, mesangial hypertrophy, proliferation, and overt proteinuria (91). Although GH actions are partially mediated through IGF-1, studies from GH transgenic mice demonstrated that alterations in the kidney and liver development are independent of IGF-1 (96). Furthermore, GH transgenic mice exhibit higher degrees of glomerular hypertrophy and podocyte damage compared to IGF-1 transgenic mice (53, 54). Conversely, GHR null diabetic mice display a substantial reduction in glomerular hypertrophy and are protected from development of DN (55). Overall, these findings indicate that elevated GH can induce glomerular hypertrophy.

## Effects of Blunting GH/GHR Axis in the Diabetic Kidney on Renal Function

On the basis of the association between elevated levels of GH and DN (2), it was hypothesized that blockade of GH action by somatostatin analogs and/or GHR antagonists might reduce the risk of diabetic renal disease. Streptozotocin-induced diabetic rats treated with octreotide, a long-acting somatostatin analog, showed reduced kidney weight and urinary albumin excretion (97). Lanreotide, another somatostatin analog, prevented diabetic renal and glomerular growth suggesting that blunting GH action could prevent experimental diabetic kidney disease (98). Administration of SMS 201-995 (a somatostatin analog) attenuated the kidney weight and ensured a significant decrease in urinary albumin excretion in experimental diabetic rats (99). GH antagonism by PTR-3173 (a somatostatin analog) elicited a blunting effect on renal hypertrophy, albuminuria, and GFR in non-obese diabetic mice (100). GHR/GH binding protein gene-disrupted mice were protected against streptozotocin-induced DN (55). Similar to somatostatin analogs, administration of GHR antagonists also elicited renoprotective effect in diabetic rodent models. GHR antagonist (G120K-PEG) ameliorated renal enlargement, glomerular hypertrophy, and urinary albumin excretion in diabetic mice (101). Furthermore, GHR antagonism offered salutary effects in non-obese diabetic mice as evidenced by normalization of kidney weight, glomerular volume, and attenuation of albuminuria (102). Therefore, modulation of GH effects may have beneficial therapeutic effects in DN.

## Summary

Podocytes are the most vulnerable component of the glomerular filtration apparatus. Podocytes have a limited capacity to divide and do not regenerate in response to injury and loss. Therefore, maintaining podocyte integrity and number is critical for normal renal function. Accumulated evidence suggests a role for the GH/GHR axis in the kidney in DN in T1DM. Studies from our group and others demonstrate that elevated levels of GH results in podocyte injury *via* multiple mechanisms including altering podocyte permselectivity, podocyte phenotypic switch, podocyte detachment, and apoptosis. In the setting of T1DM wherein elevated GH levels are associated with podocyte injury and diminished renal filtration, targeting the GH/GHR axis in podocytes could be a potential approach to combat podocytopathy. Whereas significant progress has been made in our understanding of the role of podocyte injury in DN, several questions related to podocyte repair mechanisms and regulatory pathways remain unanswered and warrant further investigation.

## Author Contributions

DM, RN, RM, and AP contributed to the review article.

## Conflict of Interest Statement

The authors declare that the research was conducted in the absence of any commercial or financial relationships that could be construed as a potential conflict of interest.
